# Monte Carlo Simulations in Nanomedicine: Advancing Cancer Imaging and Therapy

**DOI:** 10.3390/nano15020117

**Published:** 2025-01-15

**Authors:** James C. L. Chow

**Affiliations:** 1 Radiation Medicine Program, Princess Margaret Cancer Centre, University Health Network, Toronto, ON M5G 1X6, Canada; james.chow@uhn.ca; Tel.: +1-416-946-4501; 2 Department of Radiation Oncology, University of Toronto, Toronto, ON M5T 1P5, Canada

**Keywords:** nanomaterials, cancer therapy, medical imaging, Monte Carlo simulation, nanoparticle-enhanced radiotherapy, dose enhancement, DNA damage, DNA dosimetry, nanodosimetry, nanoparticle, theranostics

## Abstract

Monte Carlo (MC) simulations have become important in advancing nanoparticle (NP)-based applications for cancer imaging and therapy. This review explores the critical role of MC simulations in modeling complex biological interactions, optimizing NP designs, and enhancing the precision of therapeutic and diagnostic strategies. Key findings highlight the ability of MC simulations to predict NP bio-distribution, radiation dosimetry, and treatment efficacy, providing a robust framework for addressing the stochastic nature of biological systems. Despite their contributions, MC simulations face challenges such as modeling biological complexity, computational demands, and the scarcity of reliable nanoscale data. However, emerging technologies, including hybrid modeling approaches, high-performance computing, and quantum simulation, are poised to overcome these limitations. Furthermore, novel advancements such as FLASH radiotherapy, multifunctional NPs, and patient-specific data integration are expanding the capabilities and clinical relevance of MC simulations. This topical review underscores the transformative potential of MC simulations in bridging fundamental research and clinical translation. By facilitating personalized nanomedicine and streamlining regulatory and clinical trial processes, MC simulations offer a pathway toward more effective, tailored, and accessible cancer treatments. The continued evolution of simulation techniques, driven by interdisciplinary collaboration and technological innovation, ensures that MC simulations will remain at the forefront of nanomedicine’s progress.

## 1. Introduction

**Figure 1:**
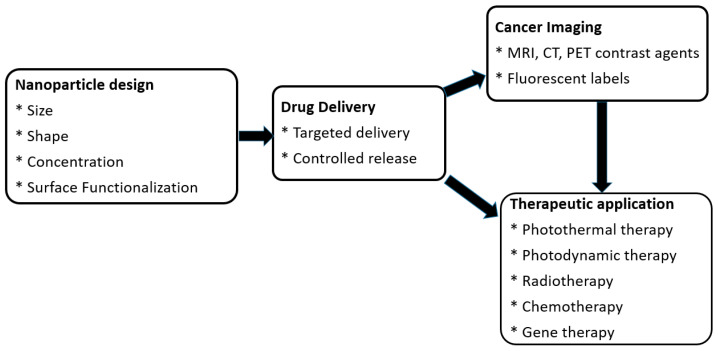
Contribution of NPs in nanomedicine for cancer imaging and therapy.

Nanoparticles (NPs) have transformed the biomedical sciences, especially in the areas of cancer imaging and therapy. Their unique physicochemical properties—such as small size, high surface area-to-volume ratio [1], and the ability to be functionalized [2]—allow for enhanced targeting, controlled drug release, and improved imaging contrast. These features make NPs ideal candidates for overcoming the limitations of conventional cancer therapies, such as poor specificity and systemic toxicity, and for advancing precision medicine [3]. In medical imaging, NP-based agents have facilitated the development of highly sensitive diagnostic tools, enabling earlier detection and better monitoring of cancer progression [4]. Similarly, in cancer therapy, they provide innovative platforms for delivering chemotherapeutic drugs, gene therapies, and even enabling modalities like photothermal and photodynamic therapy [5]. Figure 1 shows the contribution of NPs in nanomedicine, highlighting their roles in cancer imaging and therapy. It illustrates how NP design (e.g., size, shape, and surface functionalization) enables targeted drug delivery, enhances imaging (e.g., Magnetic Resonance Imaging (MRI), Computed Tomography (CT), and Positron Emission Tomography (PET) contrast agents, and fluorescent labels), and supports therapeutic applications like photothermal therapy, photodynamic therapy, radiotherapy, chemotherapy, and gene therapy.

Despite the promise of NPs, their design, optimization, and translation into clinical applications remain challenging. The interactions of NPs with biological systems are highly complex and dynamic, influenced by numerous factors including size, shape, surface charge, and the biological environment [6]. Understanding these interactions at both the cellular and systemic levels is crucial for developing effective nanomedicine strategies. However, experimental approaches alone often fall short due to time, cost, and the complexity of biological systems. This is where computational techniques, particularly Monte Carlo (MC) simulations, play a pivotal role [7].

MC simulations have emerged as a powerful computational tool in biomedical research [8], providing stochastic methods to model complex systems and predict the behavior of NPs under various conditions. These simulations are particularly valuable in studying the interactions of NPs with tissues and cells, optimizing dosimetry in cancer therapy, and improving medical imaging techniques [9]. By simulating the probabilistic nature of particle interactions, MC methods enable researchers to explore a vast parameter space, optimize NP designs, and predict outcomes that would be difficult or impossible to achieve through experimental means alone [10].

The objective of this topical review is to provide a comprehensive overview of the applications of MC simulations in the field of nanomedicine, with a focus on their role in advancing cancer imaging and therapy. We will examine the principles of MC methods, discuss their use in modeling NP behavior, and highlight studies that demonstrate their impact on improving cancer diagnostics and treatment. Beyond summarizing current advancements, this review aims to serve as a roadmap for researchers, outlining the future directions and emerging opportunities in NP research using MC simulations. By offering a prospective view, we hope to guide the development of more effective, personalized, and clinically translatable nanomedicine strategies through the continued evolution of these powerful computational tools.

## 2. MC Simulations: Fundamentals and Applications

### 2.1. Principles of MC Methods

MC methods are a class of computational algorithms that rely on repeated random sampling to obtain numerical results. These methods are particularly effective for solving problems that are deterministic in nature but analytically intractable due to their complexity [11]. MC simulations are widely used in scenarios where the solution involves a large number of variables or uncertain inputs, making direct computation impractical.

At its core, the MC method involves three fundamental steps: (1) Problem definition: The problem is framed in terms of a probabilistic model. This typically involves defining the input variables, their probability distributions, and the relationships among them; (2) Random sampling: Random samples are generated from the defined probability distributions. These samples simulate the stochastic behavior of the system under study; (3) Computation and aggregation: For each set of sampled inputs, the system’s response or outcome is calculated [12]. Repeated sampling produces a distribution of outcomes, from which statistical measures like the mean, variance, or probabilities of specific events can be estimated. MC methods excel in approximating solutions to high-dimensional integrals, optimizing complex systems, and modeling random processes [13]. The accuracy of these simulations improves with the number of samples, following the law of large numbers. However, the computational cost also scales with the number of simulations, highlighting the need for efficient sampling techniques and parallel computation [14,15].

**Figure 2:**
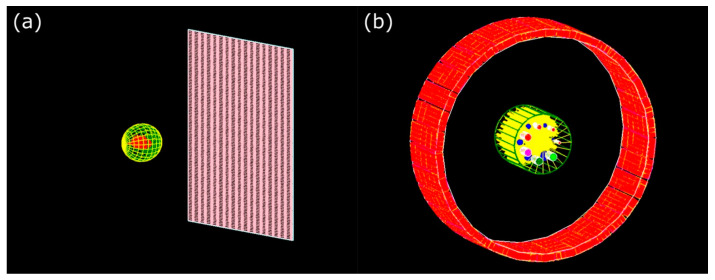
MC models of (**a**) cone-beam CT detector and Shepp–Logan phantom and (**b**) PET detection system and ACR-type phantom. Reproduced from reference [9] under the Creative Commons Attribution 4.0 International License (https://creativecommons.org/licenses/by/4.0/ (accessed on 9 December 2024)).

MC methods were developed during the 1940s, largely in response to problems encountered in nuclear physics [16]. The term “Monte Carlo” was coined by physicists Stanislaw Ulam and John von Neumann while working on the Manhattan Project [17,18], referencing the famous Monte Carlo Casino due to the method’s reliance on randomness and chance [19]. Their work was instrumental in solving neutron transport problems, which involve tracking the random interactions of particles in a medium. In physics, MC simulations have since become indispensable for studying systems with many degrees of freedom, such as molecular dynamics, statistical mechanics, and quantum field theory [20]. They provide critical insights into phenomena like phase transitions, particle behavior, and radiation transport. In medicine, MC methods are extensively used for modeling the behavior of radiation in biological tissues, which is crucial in fields such as radiation therapy [21] and medical imaging [22]. For example, in radiation therapy, MC simulations help optimize treatment plans by accurately predicting the dose distribution within the patient’s body, ensuring maximum damage to cancerous tissues while sparing healthy ones [23]. Similarly, in diagnostic imaging techniques like PET and CT scans, these simulations improve image reconstruction and enhance diagnostic accuracy [24]. Figure 2 illustrates the MC models for a cone-beam CT and PET detector system, along with their respective phantoms used for dosimetric measurements.

### 2.2. Role in NP Research

The unique properties of NPs, such as their small size, large surface area-to-volume ratio, and tunable surface chemistry, have expanded their applications across fields like medicine, environmental science, and materials engineering [25]. However, understanding their behavior in complex biological environments is challenging. MC simulations provide a valuable computational approach to model and predict NP interactions, transport, dosimetry, and distribution in these systems [26]. In biological environments, NPs interact dynamically with various entities, including cells, proteins, and other biomolecules. These interactions are influenced by particle size, shape, surface charge, and the biological milieu [27]. MC methods are particularly useful for modeling such stochastic interactions. For example, they can simulate the formation of the “protein corona”—a layer of proteins that adsorbs onto the NP surface upon entering a biological system [28,29]. The corona alters the NP’s biological identity, affecting its interactions with cells and biomolecules. MC simulations model this process by simulating protein adsorption and desorption based on binding affinities, concentrations, and NP surface properties. These models predict corona composition and dynamics, providing insights into how it influences cellular interactions and uptake. MC simulations also help model cellular uptake processes, such as receptor-mediated endocytosis, by predicting the likelihood and rates of NP internalization [30]. This information is crucial for designing NPs with optimized biological functionality.

Another critical area of NP research involves predicting their transport, distribution, and accumulation in biological systems. These processes are inherently complex, involving passive diffusion, active transport, and interactions with biological barriers such as blood vessels and cell membranes [31,32]. MC simulations enable researchers to model the random motion of NPs, including their diffusion and transport within the bloodstream. By simulating these dynamics, researchers can predict how NPs distribute across different tissues and compartments. MC methods also provide insights into the accumulation of NPs in specific tissues, such as tumors, by simulating enhanced permeability and retention (EPR) effects [33]. These predictions are vital for designing NPs that achieve targeted drug delivery and imaging with minimal off-target effects. MC simulations also offer a multi-scale modeling framework, connecting molecular-level interactions with macroscopic bio-distribution patterns [34].

**Figure 3:**
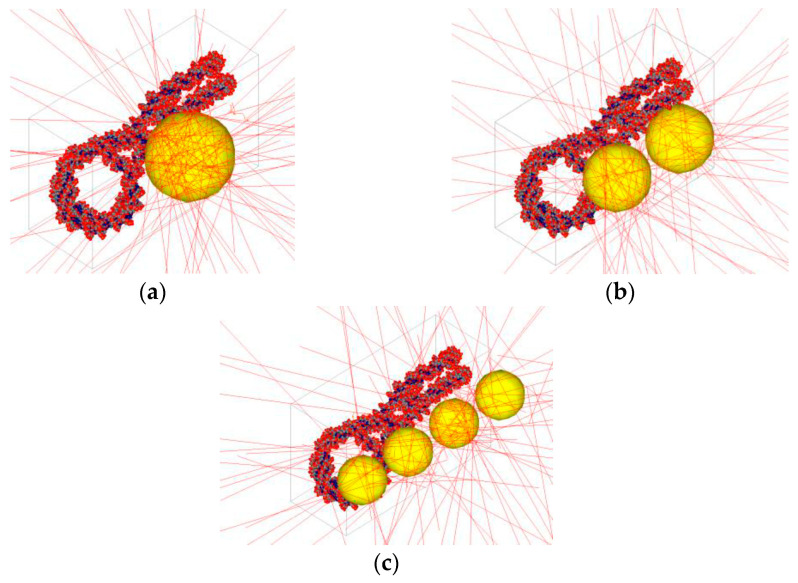
MC simulation models of irradiated single AuNPs of varying radii interacting with a DNA molecule. (**a**) shows a NP with a 5 nm radius, (**b**) shows a NP with a 3.97 nm radius, and (**c**) shows a NP with a 3.15 nm radius. The red tracks in each subfigure represent the paths of secondary electrons generated during the simulation. Reproduced from reference [39] under the Creative Commons Attribution 4.0 International License (https://creativecommons.org/licenses/by/4.0/ (accessed on 10 December 2024)).

**Figure 4:**
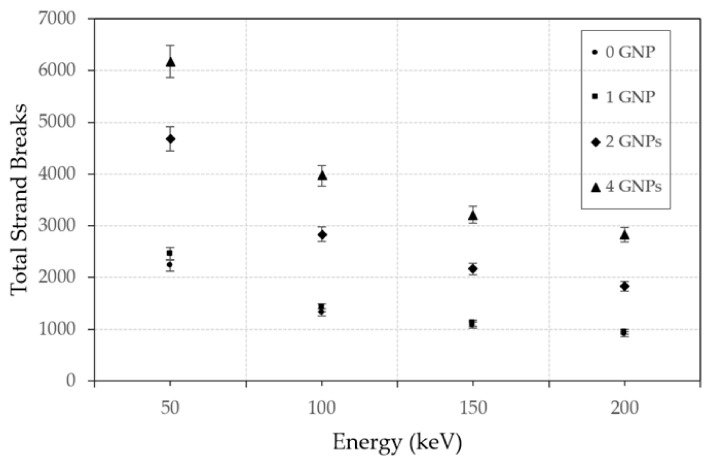
Relationship between total strand breaks and electron energy (keV) is influenced by the number of AuNPs present. Reproduced from reference [39] under the Creative Commons Attribution 4.0 International License (https://creativecommons.org/licenses/by/4.0/ (accessed on 9 December 2024)).

MC simulations play a crucial role in predicting DNA dosimetry and understanding the mechanisms of DNA damage caused by NPs in NP-enhanced radiotherapy [35,36] and imaging applications [37]. When NPs, particularly high atomic number (*Z*) materials such as gold or platinum, are introduced into biological tissues and exposed to ionizing radiation, they enhance the local radiation dose through a phenomenon known as the NP dose enhancement effect [38]. This increased dose can lead to a higher likelihood of DNA damage, including single-strand and double-strand breaks. MC simulations provide a detailed framework to model the complex interactions between radiation and matter at the molecular level [39]. Figure 3 shows the Monte Carlo simulation models of irradiated single AuNPs of different radii interacting with a DNA molecule. The simulation models include NPs with radii of 5 nm, 3.97 nm, and 3.15 nm. The red tracks in each subfigures represent the paths of secondary electrons generated during the simulation, highlighting the interaction dynamics between the NPs and the DNA molecule. Figure 4 shows the relationship between electron beam energy (50–200 keV) and the total number of strand breaks, influenced by the number of AuNPs in the simulation. The volume of gold was kept constant across all simulations, maintaining a consistent NP concentration. The results indicate that a higher number of AuNPs leads to more strand breaks, and lowering the electron beam energy further increases the strand break count [39].

By simulating the transport of radiation particles (e.g., photons, electrons) and their interactions with NPs and surrounding biological material, MC methods can accurately predict the spatial distribution of energy deposition within the cell nucleus [40]. This enables precise dosimetric calculations at the DNA scale, allowing researchers to evaluate the extent and patterns of DNA damage. Such insights are vital for optimizing NP design and radiation parameters to maximize therapeutic efficacy while minimizing collateral damage to healthy tissues.

**Figure 5:**
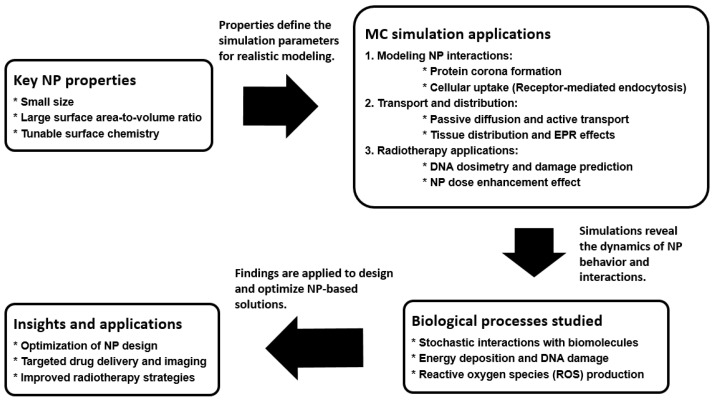
Role of MC Simulations in NP Research.

In FLASH radiotherapy, which involves ultra-high dose rate radiation delivery [41,42], MC simulations are important in understanding the generation and role of reactive oxygen species (ROS). ROS, including free radicals such as superoxide and hydroxyl radicals, are critical mediators of radiation-induced damage in cells [43]. When NPs are present, they can further enhance ROS production through secondary electron generation, leading to increased oxidative stress and cellular damage. FLASH radiotherapy has shown potential for reducing normal tissue toxicity while maintaining tumor control, largely due to its unique radiobiological effects [44]. However, the underlying mechanisms, including the interplay between ROS dynamics and ultra-high dose rates, are still under investigation. MC simulations enable researchers to model the rapid radiation-matter interactions and the subsequent ROS generation at different spatial and temporal scales. By incorporating NP-mediated effects into these models, researchers can predict how NPs influence ROS production and distribution during FLASH radiotherapy. These predictions are critical for understanding the enhanced therapeutic effects of FLASH in combination with NPs and for designing optimized treatment strategies. Figure 5 shows the role of MC simulations in NP research, highlighting their applications in modeling NP interactions, transport, and radiotherapy effects. The diagram illustrates how MC simulations provide insights into biological processes like protein corona formation, cellular uptake, and DNA damage, enabling the optimization of NP design for targeted drug delivery, imaging, and enhanced therapeutic efficacy.

### 2.3. MC NP Model

Accurate modeling of NPs in MC simulations is crucial for understanding their behavior in complex biological environments. These models must account for the physical, chemical, and biological characteristics of NPs and their interactions with cells, tissues, and biological fluids. Robust MC NP models are essential for applications in imaging, therapy, and drug delivery, enhancing efficacy and safety [7].

Key aspects of MC NP modeling include incorporating NP-specific properties like size, shape, density, atomic composition, and surface functionalization. High-*Z* materials like gold and bismuth require detailed atomic structure modeling to simulate interactions with ionizing radiation. The size and shape of NPs influence their transport properties and interaction cross-sections, critical for dose enhancement in radiotherapy or contrast generation in imaging [39]. Surface properties, including charge and functional groups, determine NP interactions with biological components, such as protein corona formation or cellular uptake [28,29].

Modeling NP interactions with biological media is critical, as these environments are highly heterogeneous. MC NP models integrate multiple layers of interaction, simulating how NPs amplify energy deposition in tissues via photoelectric and Compton effects, and cellular uptake mechanisms like receptor-mediated endocytosis and passive diffusion. Advanced MC NP models address the complexity of the tumor microenvironment, including hypoxia, vascular density, and extracellular matrix stiffness, to simulate NP distribution and accumulation in tumors accurately.

A significant advancement in NP modeling is the development of multi-scale MC models, bridging molecular, cellular, and tissue scales [32]. These models simulate radiation interactions with NPs and biomolecules at the molecular level, NP uptake and intracellular distribution at the cellular level, and NP transport and interactions within the tumor microenvironment at the tissue level. Multi-scale MC models predict therapeutic outcomes by simulating how enhanced energy deposition at the molecular scale translates into cellular damage and tumor control at the macroscopic level. They also incorporate ROS production dynamics for accurate predictions of oxidative stress-induced damage.

Despite advancements, challenges remain in modeling the dynamic and heterogeneous tumor microenvironment and incorporating biological processes like NP aggregation, clearance, and immune responses. Hybrid models combining MC methods with agent-based modeling and machine learning hold promise for addressing these challenges [37].

## 3. NPs in Biomedical Imaging

### 3.1. Imaging Modalities Enhanced by NPs

NPs have innovated biomedical imaging by enhancing the sensitivity, resolution, and specificity of various imaging modalities. Their unique physical and chemical properties, such as high Z, magnetic susceptibility, and tunable optical characteristics, make them ideal contrast agents in imaging techniques like MRI, Magnetic Particle Imaging (MPI), CT, PET, and optical imaging [45]. These advancements have significantly improved disease diagnosis, monitoring, and treatment planning.

**Figure 6:**
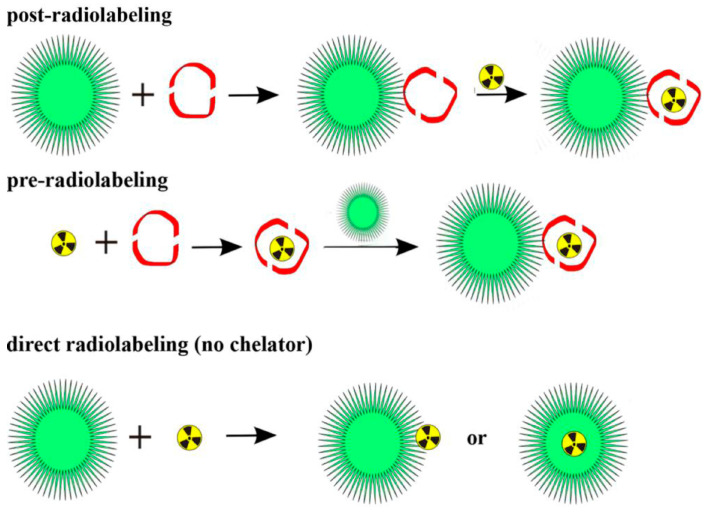
Three common radiolabeling methods involving metallic radionuclides and NPs. Reproduced from reference [49] under the Creative Commons Attribution 4.0 International License (https://creativecommons.org/licenses/by/4.0/ (accessed on 10 December 2024)).

In MRI, NPs, particularly superparamagnetic iron oxide nanoparticles (SPIONs), are employed as contrast agents to improve image contrast in soft tissues [46]. Their magnetic properties influence the relaxation times of nearby hydrogen protons, enhancing the visibility of specific tissues or pathological regions [47]. MPI, another imaging modality, leverages the unique magnetic properties of SPIONs to directly image their spatial distribution with high sensitivity and resolution, providing quantitative and real-time imaging capabilities without background noise from biological tissues. Similarly, in CT, high-Z NPs, such as gold or bismuth, serve as contrast agents by amplifying X-ray attenuation. This results in higher-contrast images with improved differentiation between tissues, enabling more precise detection of tumors and vascular abnormalities [48]. PET imaging benefits from NPs functionalized with positron-emitting isotopes. These radiolabeled NPs not only enhance the imaging signal but also provide a platform for targeted imaging, where NPs are directed to specific cells or tissues using ligands or antibodies [49]. Figure 6 shows three radiolabeling techniques using metallic radionuclides like Cu-64, Ga-68, and Zr-89 with NPs. These techniques are post-radiolabeling, pre-radiolabeling, and direct radiolabeling. This specificity improves the detection of cancerous lesions and metastases. In optical imaging, quantum dots and other fluorescent NPs offer superior brightness and photostability compared to traditional dyes, enabling the real-time imaging of molecular and cellular processes with high spatial resolution [50].

MC simulations have been pivotal in optimizing the use of NPs across these imaging modalities. By simulating complex interactions between particles, tissues, and imaging systems, MC methods enable the evaluation of imaging performance under various conditions. For example, in CT imaging, MC simulations help optimize NP concentration and distribution to maximize contrast while minimizing radiation dose [51,52]. In PET, simulations aid in determining optimal isotope loading on NPs for improved signal-to-noise ratios and reduced patient exposure to radioactivity [53]. Case studies have demonstrated the power of MC simulations in refining imaging strategies. For example, in MRI, simulations have been used to model the distribution of SPIONs within tumors, providing insights into their impact on image contrast and guiding the development of more efficient NP formulations [54,55]. Similarly, in optical imaging, MC simulations have helped optimize light–tissue interactions, improving the accuracy of fluorescence-based imaging techniques [56,57].

### 3.2. MC Simulation in Image Reconstruction

**Figure 7:**
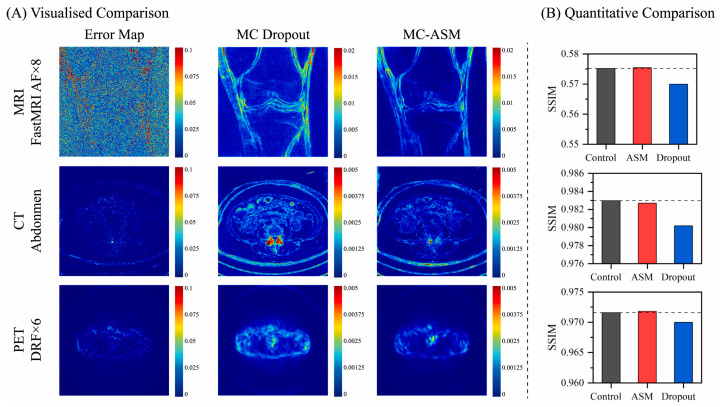
(**A**) Visualized samples of uncertainty maps generated by the MC dropout and MC-ASM, along with the corresponding error maps. (**B**) Quantitative comparison between (1) Arbitrary-Masked Mamba-based model (MambaMIR) without MC-ASM or MC dropout (control group), (2) MambaMIR with MC-ASM, and (3) MambaMIR with MC dropout across three datasets. Reproduced from reference [58] under the Creative Commons Attribution 4.0 International License (https://creativecommons.org/licenses/by/4.0/ (accessed on 10 December 2024)).

NPs have enhanced biomedical imaging by offering unique optical, magnetic, and radiative properties that innovate imaging modalities such as fluorescence imaging, MRI, and CT. However, the accurate reconstruction of images involving NPs requires sophisticated modeling to account for their complex interactions with light, radiation, and biological tissues. MC simulations have emerged as a tool for tackling these challenges [58]. Figure 7 shows the outcomes of various MC methods in image reconstruction. Two MC techniques, namely, common MC dropout and the innovative MC-Arbitrary Scan Masking (ASM), enhanced image reconstructions of MRI, CT, and PET compared to the control groups. The MC-ASM algorithm is a novel approach that combines MC sampling with a dynamic scan-masking strategy to selectively mask input data, thereby simulating arbitrary sampling patterns. This method integrates with deep learning models to account for variability in scan data, allowing for more robust and accurate reconstructions even under sparse or incomplete sampling conditions. Notably, the MC-ASM method, leveraging its ability to mimic real-world noise and data irregularities, delivered the best performance, achieving superior image quality and resolution across imaging modalities. The Structural Similarity Index Measure (SSIM) in Figure 7B is a metric that evaluates the quality of an image by comparing its structural, luminance, and contrast similarities to a reference image. The dashed line in Figure 7B indicates the maximum SSIM value in the plot. Unlike traditional metrics like Mean Squared Error (MSE), SSIM aligns closely with human visual perception, with values ranging from −1 to 1, where 1 indicates perfect similarity.

NPs can enhance imaging signals, but noise from scattering, absorption, and background interference often reduces the clarity of reconstructed images. MC simulations provide a robust framework for modeling these interactions at a microscopic level, enabling the separation of NP-specific signals from background noise. For example, in fluorescence imaging, MC simulations accurately model the scattering of emitted photons in tissues, improving signal detection and localization [59]. When coupled with NP-based fluorescent probes, this approach achieves a higher signal-to-noise ratio, critical for detecting tumors at early stages [60]. In photoacoustic imaging, NPs such as gold nanorods serve as contrast agents by efficiently converting absorbed light into acoustic waves [61]. MC simulations help optimize the laser parameters and NP distribution to maximize signal strength while minimizing noise, resulting in clearer reconstructed images [62]. These simulations are particularly valuable in scenarios where noise dominates due to tissue heterogeneity or low NP concentrations.

The unique physical properties of NPs, such as their ability to selectively accumulate in tumor tissues (via enhanced permeability and retention effects), inherently enhance imaging contrast. MC simulations amplify this advantage by providing precise models of NP behavior in biological environments. For example, in MRI, SPIONs serve as contrast agents by altering the local magnetic field [63]. MC-based models simulate these magnetic perturbations to refine image reconstruction algorithms, thereby improving contrast between tumor and healthy tissues. Similarly, in CT imaging, NPs like gold exhibit high X-ray attenuation coefficients, offering superior contrast compared to traditional agents. MC simulations model the intricate interactions between X-rays, NPs, and tissues, enabling enhanced resolution and structural delineation in reconstructed images [64]. These advancements allow for the visualization of microvascular details and early-stage tumor boundaries that would otherwise be undetectable.

## 4. NPs in Cancer Therapy

### 4.1. Radiotherapy and Radiosensitization

Radiotherapy is a cornerstone of cancer treatment, using ionizing radiation to damage the DNA of cancer cells and inhibit their proliferation [65]. However, the efficacy of radiotherapy can be limited by the inherent radioresistance of tumors and the collateral damage to surrounding healthy tissues [66]. The introduction of NPs, particularly gold nanoparticles (AuNPs), has transformed radiotherapy by acting as potent radiosensitizers. These NPs amplify radiation effects within tumors, offering enhanced therapeutic outcomes [67]. MC simulations play a pivotal role in optimizing the use of AuNPs in radiotherapy, particularly for dosimetry and treatment planning.

AuNPs have emerged as highly effective radiosensitizers due to their unique physical and chemical properties. Their high atomic number (*Z* = 79) results in a significant photoelectric absorption cross-section, particularly under low-energy X-rays, which leads to the generation of secondary electrons, Auger electrons, and free radicals [68]. These reactive species cause localized damage to cancer cells, enhancing the therapeutic efficacy of radiation treatments. In addition to their physical properties, AuNPs can be functionalized with targeting ligands, such as antibodies or peptides, to selectively accumulate in tumors via active targeting mechanisms [5,69]. This selective accumulation minimizes radiation exposure to healthy tissues, reducing side effects. Studies have shown that AuNPs enhance the radiation dose delivered to tumors by up to several fold, particularly in photon-based therapies [70].

**Figure 8:**
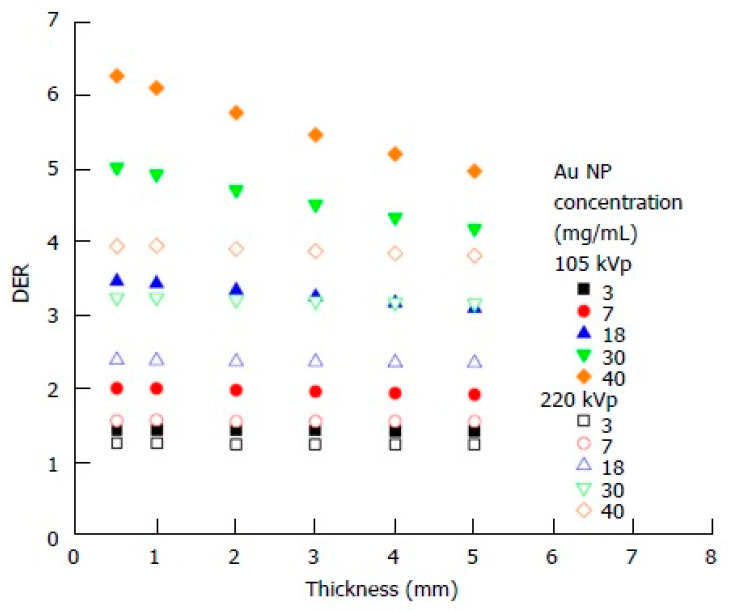
Relationship between the dose enhancement ratio (DER) and skin target thickness with varying concentrations of AuNPs using 105 and 220 kVp photon beams. Reproduced from reference [74] under the Creative Commons Attribution 4.0 International License (https://creativecommons.org/licenses/by/4.0/ (accessed on 10 December 2024)).

The precise application of AuNPs in radiotherapy relies heavily on accurate dosimetry and treatment planning. MC simulations are vital in this context, as they provide a detailed stochastic modeling of radiation transport and interactions within tissues containing NPs [71]. These simulations account for the physical properties of AuNPs, including their size, shape, concentration, and spatial distribution, as well as their interaction with different radiation types, such as photons, electrons, or protons. MC-based nanodosimetry enables the quantification of enhanced dose deposition in tumor tissues due to the presence of AuNPs [72]. For example, MC simulations have demonstrated that NP clusters amplify dose heterogeneity within tumors, offering insights into optimizing radiation delivery [73]. Furthermore, these simulations assist in identifying optimal radiation energies that maximize the NP-induced dose enhancement while minimizing damage to surrounding healthy tissues [74]. In treatment planning, MC methods integrate biological factors, such as the radiosensitivity of tumors and the oxygenation status, with NP distributions to develop personalized radiotherapy protocols [75]. This capability allows for precise tailoring of radiation dose and beam configuration to maximize therapeutic benefits. Figure 8 shows the relationship between the dose enhancement ratio (DER) and skin target thickness, taking into account different AuNP concentrations, with 105 and 220 kVp photon beams. The AuNP concentration is chosen based on the levels used in small-animal experiments. It reveals that, for 220 kVp photon beams, the dose enhancement factor remains largely unaffected by skin target thickness when AuNP concentrations range from 3 to 40 mg/mL. Conversely, for 105 kVp photon beams, the enhancement factor increases as the target thickness decreases, especially when the NP concentration is above 18 mg/mL [74].

### 4.2. Photothermal and Photodynamic Therapy

Photothermal therapy (PTT) and photodynamic therapy (PDT) are cutting-edge cancer treatments that utilize the unique interactions between light and NPs to achieve localized tumor destruction. In PTT, NPs absorb near-infrared (NIR) light and convert it into heat, leading to thermal ablation of cancer cells. In PDT, light activation of NPs or photosensitizers generates ROS, which cause oxidative damage to tumor tissues. Both therapies benefit immensely from computational modeling and simulation, particularly for optimizing thermal effects, ROS generation, and dose delivery [76]. MC simulations play a critical role in these processes by providing insights into light–tissue interactions and the spatial–temporal dynamics of treatment.

The effectiveness of PTT hinges on the ability to generate sufficient heat to cause irreversible tumor cell damage while sparing surrounding healthy tissues. AuNPs, carbon-based nanostructures, and other plasmonic materials are widely employed in PTT due to their efficient light absorption and photothermal conversion properties [77]. To predict and optimize thermal effects, MC simulations model the transport of light in heterogeneous biological tissues, considering scattering, absorption, and NP distribution. Simulations also account for heat diffusion dynamics, enabling precise predictions of temperature elevation in the targeted region [78]. These models incorporate factors such as laser wavelength, power density, NP concentration, and tumor geometry to achieve controlled and uniform thermal ablation. In PDT, the generation of ROS, particularly singlet oxygen, is the primary mechanism for inducing tumor cell apoptosis. MC simulations model the propagation and absorption of light within tissues, ensuring the sufficient activation of photosensitizers localized in tumors [79]. Moreover, these simulations predict the spatial distribution of ROS and their diffusion in tissue microenvironments, which is critical for maximizing therapeutic effects while minimizing off-target oxidative damage [80].

**Figure 9:**
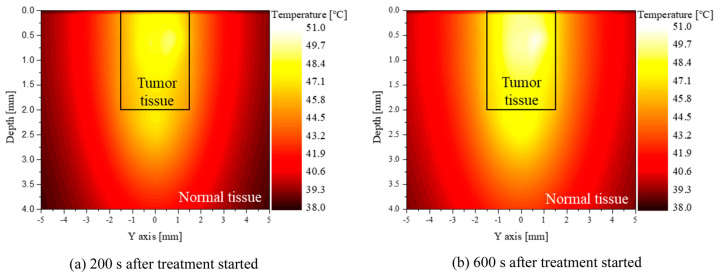
Temperature distribution of the medium (φ_drr_ = 1, 3 AuNP injections, Pl = 50 mW). Reproduced from reference [81] under the Creative Commons Attribution 4.0 International License (https://creativecommons.org/licenses/by/4.0/ (accessed on 10 December 2024)).

The success of PTT and PDT depends on precise dose and temperature optimization. MC simulations assist in determining the ideal light dosimetry for NP activation. In PTT, these simulations guide the selection of laser parameters, such as fluence rate and exposure duration, to achieve tumor-specific heating thresholds without causing thermal damage to adjacent healthy tissues [81]. Figure 9 shows the temperature distribution in the YZ plane of normal and tumor tissue at 200 and 600 s after laser irradiation, with the distribution radius ratio of AuNPs (φ_drr_) = 1, three injections, and a laser power of 50 mW at 1064 nm. The black box indicates tumor tissue. After 600 s, the temperature was higher than at 200 s. Approximately 96% of the tumor volume reached the apoptosis temperature range at 200 s, and 94% at 600 s. However, normal tissue beneath the tumor reached around 48.5 °C after 600 s, risking thermal damage [81].

For PDT, MC-based models integrate photosensitizer photophysics, tissue oxygenation, and light propagation to optimize the generation and diffusion of ROS. Simulation studies reveal the influence of tumor hypoxia—a common challenge in PDT—on treatment efficacy and help design strategies such as fractionated light delivery or oxygen supplementation to overcome these limitations [82]. Furthermore, hybrid PTT-PDT approaches, which combine the thermal and oxidative effects of NPs, have gained attention for synergistic cancer therapy [83]. MC simulations are instrumental in evaluating the interplay between heat generation and ROS dynamics, enabling comprehensive treatment planning that advantages the strengths of both modalities.

## 5. Synergistic Applications

**Figure 10:**
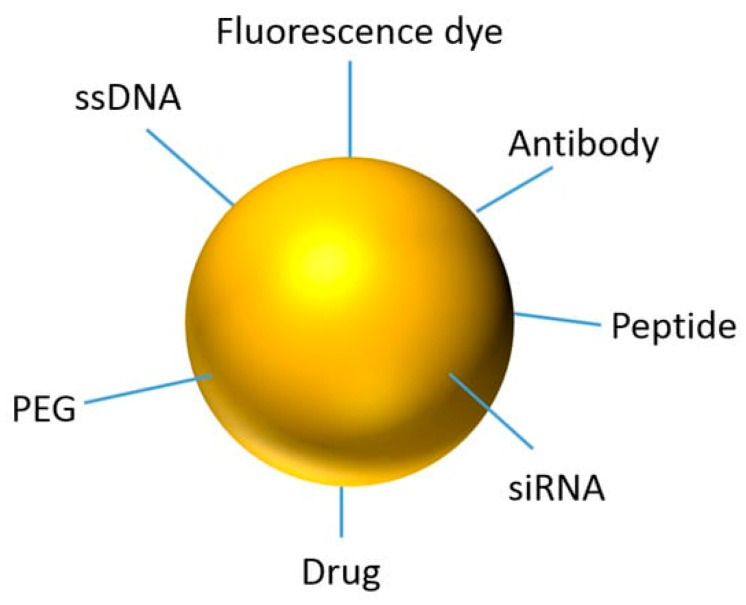
Depiction of an AuNP for theranostic applications. Reproduced from reference [5] under the Creative Commons Attribution 4.0 International License (https://creativecommons.org/licenses/by/4.0/ (accessed on 10 December 2024)).

The integration of imaging and therapeutic functionalities into single NP systems, known as theranostics, represents a transformative approach in nanomedicine [84]. Theranostic NPs enable simultaneous cancer diagnosis, the real-time monitoring of therapeutic delivery, and effective treatment, creating opportunities for personalized medicine [5]. Figure 10 shows that various functional groups, including PEG, ssDNA, antibodies, peptides, drugs, fluorescence markers, and siRNA, can be attached to the particle surface to enable both imaging and therapeutic functions in cancer nanotheranostics.

MC simulations play an important role in optimizing the design and application of dual-function NP systems, ensuring their efficacy in both imaging and therapy [85]. Theranostic NPs combine diagnostic imaging modalities such as MRI, CT, or fluorescence imaging with therapeutic functions like PTT, PDT, or drug delivery. For example, AuNPs functionalized with fluorescent dyes can enable high-resolution tumor imaging while simultaneously inducing photothermal ablation upon NIR light exposure [86]. Similarly, iron oxide NPs can serve as contrast agents for MRI and be employed for hyperthermia-based cancer therapy [47,63]. One key advantage of theranostic NPs is their ability to localize and act specifically within tumor tissues through passive mechanisms (e.g., EPR effect) or active targeting via ligand-receptor interactions. This targeted dual functionality enhances therapeutic efficacy while minimizing off-target effects [87].

MC simulations are essential in the development and optimization of dual-function NP systems. By modeling the complex interactions of NPs with biological tissues, radiation, and light, MC simulations provide insights into the behavior of theranostic agents under various physiological and therapeutic conditions. In imaging, MC simulations help evaluate the signal generation capabilities of NPs across different modalities. For example, in fluorescence imaging, MC models account for light scattering, absorption, and NP emission, optimizing the contrast and resolution [88]. In CT or MRI, simulations analyze NP interactions with X-rays or magnetic fields to enhance image quality while minimizing exposure to ionizing radiation or optimizing magnetic field parameters [89]. For therapeutic applications, MC simulations model the deposition of energy within tumor tissues, whether from thermal energy in PTT, ROS in PDT, or radiation in NP-enhanced radiotherapy [90]. In dual-function systems, these simulations address the interplay between imaging and therapeutic mechanisms, ensuring that the NPs perform both roles effectively without compromising one for the other [91]. Furthermore, MC studies enable the optimization of NP properties such as size, shape, composition, and functionalization. They also provide insights into the bio-distribution and clearance of theranostic agents, ensuring safety and efficacy in clinical applications. For example, MC simulations can guide the design of NPs with optimal optical or radiative properties for imaging while maintaining the necessary energy absorption characteristics for therapy [92].

## 6. Challenges and Future Directions

MC simulations have been instrumental in advancing cancer imaging and therapy by providing detailed insights into the complex interactions of NPs within biological systems. However, several challenges continue to limit their effectiveness, and emerging trends and technologies present opportunities for significant advancements. One of the major challenges in MC simulation lies in modeling the intricate biological complexity of the tumor microenvironment [93]. Tumors exhibit heterogeneous cellular structures, dynamic vasculature, and immune interactions that are difficult to replicate with current simulation capabilities [94]. Simplifications in models can lead to discrepancies between simulations and real-world biological behavior. In addition, the scarcity of reliable biological and physicochemical data at the nanoscale hinders accurate calibration and validation of simulations. Computational intensity remains a significant barrier, as MC simulations require vast numbers of iterations to produce statistically robust results, which is particularly demanding for high-resolution geometries or multiphysics modeling [95]. Bridging the gaps between disciplines, such as computational science, biology, and physics, is another ongoing challenge that limits the practical application of MC simulations in nanomedicine.

Recent advances in NP design, simulation technology, and novel methodologies are beginning to address these challenges. Hybrid models that integrate MC methods with deterministic approaches, such as finite element methods or machine learning algorithms, offer a way to simulate complex systems more efficiently [96]. In parallel, FLASH radiotherapy—a novel technique that delivers ultra-high-dose radiation in extremely short bursts—has emerged as a promising cancer therapy [41,42]. MC simulations are being adapted to model the unique physical and biological effects of FLASH radiotherapy, including its ability to spare normal tissues while maintaining tumor control [97,98]. This requires MC simulations to evolve to capture ultrafast dynamics and high-dose deposition patterns with unprecedented precision.

Another important development is the application of quantum computing to MC simulations. Quantum algorithms have the potential to drastically reduce computational time by efficiently solving complex probabilistic problems inherent in MC methods [99]. By harnessing quantum superposition and entanglement, researchers could simulate larger systems at atomic and molecular scales, enabling more detailed studies of NP interactions within biological environments. Quantum computing could also facilitate the development of patient-specific simulations by processing vast datasets, such as those derived from multi-omics and imaging studies, much faster than classical computers [100].

The integration of big data and high-performance computing further enhances the clinical relevance of MC simulations. Advances in artificial intelligence and machine learning allow simulations to incorporate large-scale experimental datasets for model refinement [101]. This is particularly valuable for designing multifunctional NPs with specific therapeutic and diagnostic properties. Simulations are also increasingly being used to predict the behavior of these NPs in personalized scenarios, optimizing their bio-distribution, dosimetry, and therapeutic efficacy [102].

The translation of these advancements to clinical applications offers exciting opportunities for personalized nanomedicine. MC simulations can guide the design of patient-specific treatments, tailoring NP formulations to optimize outcomes while minimizing side effects. For example, integrating FLASH radiotherapy with personalized dosimetry could revolutionize cancer therapy by providing highly effective treatments with reduced toxicity [103]. Furthermore, MC simulations could streamline clinical trials by predicting outcomes and optimizing protocols, reducing the need for extensive preclinical testing. These simulations also hold potential for aiding regulatory processes by providing robust safety and efficacy data for emerging nanotherapies [104,105]. Table 1 outlines the key limitations of current MC simulation techniques in cancer imaging and therapy, alongside emerging technologies and methodologies aimed at addressing these challenges to enhance clinical translation and personalized nanomedicine.

## 7. Conclusions

MC simulations have emerged as powerful tools in advancing NP-based applications for cancer imaging and therapy. This review highlights the pivotal role of MC simulations in unraveling complex biological interactions, optimizing NP design, and enhancing treatment precision. By providing a probabilistic framework to model the stochastic nature of NP behavior in biological systems, MC simulations have become indispensable for simulating processes such as drug delivery, radiation dosimetry, and bio-distribution with remarkable accuracy. Despite current limitations, including challenges in modeling biological complexity, computational intensity, and data scarcity, MC simulations continue to drive progress in nanomedicine. Emerging technologies, such as hybrid modeling approaches, high-performance computing, and quantum algorithms, promise to overcome these barriers. Furthermore, innovations like FLASH radiotherapy and multifunctional theranostic NPs are pushing the boundaries of MC simulations, enabling the exploration of novel therapeutic paradigms and clinical applications. The transformative potential of MC simulations lies in their ability to bridge fundamental research and clinical practice. As these simulations become increasingly integrated with patient-specific data, they offer the possibility of personalized nanomedicine, where treatments are tailored to individual physiological and genetic profiles. Moreover, their role in streamlining clinical trials and supporting regulatory approval processes underscores their importance in accelerating the translation of nanotechnology-based therapies to the clinic.

In conclusion, MC simulations represent a cornerstone of modern nanomedicine, with the capacity to revolutionize cancer diagnostics and therapy. Continued advancements in simulation techniques, interdisciplinary collaboration, and integration with emerging technologies will ensure that MC simulations remain at the forefront of innovation, paving the way for more precise, effective, and accessible healthcare solutions.

## Figures and Tables

**Table 1:** Summary of challenges and future directions for MC simulations in nanomedicine.

Aspect	Challenges	Future Directions
Modeling Biological Complexity	Simplified representations of tumor microenvironments lead to inaccuracies. Limited understanding of dynamic vasculature and immune responses.	Develop high-fidelity models incorporating dynamic and heterogeneous biological interactions. Leverage experimental data from advanced imaging and omics technologies for model refinement.
Data Scarcity and Quality	Lack of reliable physicochemical and biological data at the nanoscale hinders calibration. Incomplete datasets limit validation.	Integrate large-scale datasets (e.g., multi-omics, imaging) for improved accuracy. Use artificial intelligence and machine learning to bridge data gaps and improve prediction accuracy.
Computational Intensity	High computational demands for detailed simulations. Difficulty modeling complex geometries and ultrafast dynamics in a feasible timeframe.	Adopt quantum computing to enhance computational efficiency. Utilize hybrid models combining MC simulations with deterministic methods or artificial intelligence algorithms. Leverage high-performance and cloud computing.
Emerging Treatment Techniques	Lack of established simulation methods for novel therapies like FLASH radiotherapy. Difficulty adapting existing models to capture ultrafast dynamics.	Develop specialized MC simulation frameworks to model FLASH radiotherapy. Incorporate ultrafast radiation dose deposition and biological response dynamics into simulations.
NP Design	Challenges in predicting interactions of multifunctional or novel NPs. Limited capability to simulate complex theranostic agents.	Simulate advanced NP designs, including multifunctional and biodegradable systems. Incorporate molecular-level interactions to refine predictions.
Clinical Translation	Bridging the gap between simulation outcomes and real-world applicability remains difficult. Regulatory hurdles due to limited simulation validation for clinical use.	Use patient-specific data to guide personalized treatment designs. Streamline clinical trials by using MC simulations for prediction and optimization. Support regulatory approval with robust data.
Interdisciplinary Gaps	Limited collaboration among computational scientists, biologists, and physicists. Knowledge silos hinder integrated approaches to complex problems.	Foster interdisciplinary research collaborations. Develop standardized frameworks and protocols for MC simulations in nanomedicine.

## Data Availability

No new data was created.

## References

[1] Alexis F., Pridgen E.M., Langer R., Farokhzad O.C. (2010). Nanoparticle technologies for cancer therapy. Drug Deliv..

[2] Subbiah R., Veerapandian M., Yun K.S. (2010). Nanoparticles: Functionalization and multifunctional applications in biomedical sciences. Curr. Med. Chem..

[3] Mitchell M.J., Billingsley M.M., Haley R.M., Wechsler M.E., Peppas N.A., Langer R. (2021). Engineering precision nanoparticles for drug delivery. Nat. Rev. Drug Discov..

[4] Thierry B. (2009). Drug nanocarriers and functional nanoparticles: Applications in cancer therapy. Curr. Drug Deliv..

[5] Siddique S., Chow J.C.L. (2020). Gold nanoparticles for drug delivery and cancer therapy. Appl. Sci..

[6] Al-Thani A.N., Jan A.G., Abbas M., Geetha M., Sadasivuni K.K. (2024). Nanoparticles in cancer theragnostic and drug delivery: A comprehensive review. Life Sci..

[7] Chow J.C.L. (2018). Recent progress in Monte Carlo simulation on gold nanoparticle radiosensitization. AIMS Biophys..

[8] Velikova T., Mileva N., Naseva E. (2024). Method “Monte Carlo” in healthcare. World J. Methodol..

[9] Lee H. (2024). Monte Carlo methods for medical imaging research. Biomed. Eng. Lett..

[10] Li W.B., Müllner M., Greiter M.B., Bissardon C., Xie W.Z., Schlatll H., Oeh U., Li J.L., Hoeschen C. (2014). Monte Carlo simulations of dose enhancement around gold nanoparticles used as x-ray imaging contrast agents and radiosensitizers. Medical Imaging 2014, Physics of Medical Imaging.

[11] Harrison R.L. (2010). Introduction to Monte Carlo simulation. AIP Conf. Proc..

[12] Rubinstein R.Y., Kroese D.P. (2016). Simulation and the Monte Carlo Method.

[13] Bonate P.L. (2001). A brief introduction to Monte Carlo simulation. Clin. Pharmacokinet..

[14] Wu F., Dantan J.Y., Etienne A., Siadat A., Martin P. (2009). Improved algorithm for tolerance allocation based on Monte Carlo simulation and discrete optimization. Comput. Ind. Eng..

[15] Alerstam E., Svensson T., Andersson-Engels S. (2008). Parallel computing with graphics processing units for high-speed Monte Carlo simulation of photon migration. J. Biomed. Opt..

[16] Bielajew A.F. (2021). History of monte carlo. Monte Carlo Techniques in Radiation Therapy.

[17] Los Alamos National Laboratory Hitting the Jackpot: The Birth of the Monte Carlo Method. https://www.lanl.gov/media/publications/actinide-research-quarterly/first-quarter-2023/hitting-the-jackpot-the-birth-of-the-monte-carlo-method.

[18] Benov D.M. (2016). The Manhattan Project, the first electronic computer and the Monte Carlo method. Monte Carlo Methods Appl..

[19] Metropolis N. (1987). The beginning of the Monte Carlo method. Los Alamos Sci..

[20] Carlson J., Gandolfi S., Pederiva F., Pieper S.C., Schiavilla R., Schmidt K.E., Wiringa R.B. (2015). Quantum Monte Carlo methods for nuclear physics. Rev. Mod. Phys..

[21] Rogers D.W. (2006). Fifty years of Monte Carlo simulations for medical physics. Phys. Med. Biol..

[22] Zaidi H. (1999). Relevance of accurate Monte Carlo modeling in nuclear medical imaging. Med. Phys..

[23] Brualla L., Rodriguez M., Lallena A.M. (2017). Monte Carlo systems used for treatment planning and dose verification. Strahlenther. Onkol..

[24] Saaidi R., Zeghari A., El Moursli R.C. (2024). Monte Carlo simulation of two Siemens biograph PET/CT system using gate: Image quality performance. Radiat. Phys. Chem..

[25] Rudramurthy G.R., Swamy M.K. (2018). Potential applications of engineered nanoparticles in medicine and biology: An update. J. Biol. Inorg. Chem..

[26] Moradi F., Saraee K.R., Sani S.A., Bradley D.A. (2021). Metallic nanoparticle radiosensitization: The role of Monte Carlo simulations towards progress. Radiat. Phys. Chem..

[27] Mailander V., Landfester K. (2009). Interaction of nanoparticles with cells. Biomacromolecules.

[28] Zhdanov V.P. (2019). Formation of a protein corona around nanoparticles. Curr. Opin. Colloid. Interface Sci..

[29] Carnal F., Clavier A., Stoll S. (2016). Polypeptide-nanoparticle interactions and corona formation investigated by monte carlo simulations. Polymers.

[30] Deng H., Dutta P., Liu J. (2019). Stochastic modeling of nanoparticle internalization and expulsion through receptor-mediated transcytosis. Nanoscale.

[31] Liu H.H., Surawanvijit S., Rallo R., Orkoulas G., Cohen Y. (2011). Analysis of nanoparticle agglomeration in aqueous suspensions via constant-number Monte Carlo simulation. Environ. Sci. Technol..

[32] Shi X., Tian F. (2019). Multiscale modeling and simulation of nano-carriers delivery through biological barriers—A review. Adv. Theory Simul..

[33] Peukert D., Kempson I., Douglass M., Bezak E. (2019). Gold nanoparticle enhanced proton therapy: Monte Carlo modeling of reactive species’ distributions around a gold nanoparticle and the effects of nanoparticle proximity and clustering. Int. J. Mol. Sci..

[34] Jiang L., Guo Y., Liu Z., Chen S. (2024). Computational understanding of the coalescence of metallic nanoparticles: A mini review. Nanoscale.

[35] Chow J.C.L. (2024). Biophysical insights into nanomaterial-induced DNA damage: Mechanisms, challenges, and future directions. AIMS Biophys..

[36] Berbeco R.I., Korideck H., Ngwa W., Kumar R., Patel J., Sridhar S., Johnson S., Price B.D., Kimmelman A., Makrigiorgos G.M. (2012). DNA damage enhancement from gold nanoparticles for clinical MV photon beams. Radiat. Res..

[37] Kirillin M., Shirmanova M., Sirotkina M., Bugrova M., Khlebtsov B., Zagaynova E. (2009). Contrasting properties of gold nanoshells and titanium dioxide nanoparticles for optical coherence tomography imaging of skin: Monte Carlo simulations and in vivo study. J. Biomed. Opt..

[38] Díaz-Galindo C.A., Garnica-Garza H.M. (2024). Gold nanoparticle-enhanced radiotherapy: Dependence of the macroscopic dose enhancement on the microscopic localization of the nanoparticles within the tumor vasculature. PLoS ONE.

[39] Santiago C.A., Chow J.C.L. (2023). Variations in Gold Nanoparticle Size on DNA Damage: A Monte Carlo Study Based on a Multiple-Particle Model Using Electron Beams. Appl. Sci..

[40] Antunes J., Pinto C.I., Campello M.P., Santos P., Mendes F., Paulo A., Sampaio J.M. (2024). Utility of realistic microscopy-based cell models in simulation studies of nanoparticle-enhanced photon radiotherapy. Biomed. Phys. Eng. Express.

[41] Chow J.C.L., Ruda H.E. (2023). Flash Radiotherapy: Innovative Cancer Treatment. Encyclopedia.

[42] Siddique S., Ruda H.E., Chow J.C.L. (2023). FLASH Radiotherapy and the Use of Radiation Dosimeters. Cancers.

[43] Shiraishi Y., Matsuya Y., Fukunaga H. (2024). Possible mechanisms and simulation modeling of FLASH radiotherapy. Radiol. Phys. Technol..

[44] Chow J.C.L., Ruda H.E. (2024). Mechanisms of Action in FLASH Radiotherapy: A Comprehensive Review of Physicochemical and Biological Processes on Cancerous and Normal Cells. Cells.

[45] Padmanabhan P., Kumar A., Kumar S., Chaudhary R.K., Gulyás B. (2016). Nanoparticles in practice for molecular-imaging applications: An overview. Acta Biomater..

[46] Antonelli A., Magnani M. (2022). SPIO nanoparticles and magnetic erythrocytes as contrast agents for biomedical and diagnostic applications. J. Magn. Magn. Mater..

[47] Chow J.C.L., Wu K., Wang J.-P. (2024). Magnetic Nanoparticles in Magnetic Resonance Imaging: Principles and Applications. Magnetic Nanoparticles in Nanomedicine.

[48] Cormode D.P., Naha P.C., Fayad Z.A. (2014). Nanoparticle contrast agents for computed tomography: A focus on micelles. Contrast Media Mol. Imaging.

[49] Stockhofe K., Postema J.M., Schieferstein H., Ross T.L. (2014). Radiolabeling of nanoparticles and polymers for PET imaging. Pharmaceuticals.

[50] Wu Y., Ali M.R., Chen K., Fang N., El-Sayed M.A. (2019). Gold nanoparticles in biological optical imaging. Nano Today.

[51] Jones B.L., Cho S.H. (2011). The feasibility of polychromatic cone-beam x-ray fluorescence computed tomography (XFCT) imaging of gold nanoparticle-loaded objects: A Monte Carlo study. Phys. Med. Biol..

[52] Dou Y., Guo Y., Li X., Li X., Wang S., Wang L., Lv G., Zhang X., Wang H., Gong X. (2016). Size-tuning ionization to optimize gold nanoparticles for simultaneous enhanced CT imaging and radiotherapy. ACS Nano.

[53] Cho J., Wang M., Gonzalez-Lepera C., Mawlawi O., Cho S.H. (2016). Development of bimetallic (Zn@ Au) nanoparticles as potential PET-imageable radiosensitizers. Med. Phys..

[54] Maschmeyer R.T., Gholami Y.H., Kuncic Z. (2020). Clustering effects in nanoparticle-enhanced β− emitting internal radionuclide therapy: A Monte Carlo study. Phys. Med. Biol..

[55] Martin É., Gossuin Y., Bals S., Kavak S., Vuong Q.L. (2022). Monte Carlo simulations of the magnetic behaviour of iron oxide nanoparticle ensembles: Taking size dispersion, particle anisotropy, and dipolar interactions into account. Eur. Phys. J. B.

[56] Lopushenko I., Sieryi O., Bykov A., Meglinski I. (2024). Exploring the evolution of circular polarized light backscattered from turbid tissue-like disperse medium utilizing generalized Monte Carlo modeling approach with a combined use of Jones and Stokes–Mueller formalisms. J. Biomed. Opt..

[57] Yang X., Chai C., Zuo H., Chen Y.H., Shi J., Ma C., Sawan M. (2024). Monte Carlo-Based Optical Simulation of Optical Distribution in Deep Brain Tissues Using Sixteen Optical Sources. Bioengineering.

[58] Huang J., Yang L., Wang F., Wu Y., Nan Y., Wu W., Wang C., Shi K., Aviles-Rivero A.I., Schönlieb C.B. (2025). Enhancing global sensitivity and uncertainty quantification in medical image reconstruction with Monte Carlo arbitrary-masked mamba. Med. Image Anal..

[59] Cong A.X., Hofmann M.C., Cong W., Xu Y., Wang G. (2011). Monte Carlo fluorescence microtomography. J. Biomed. Opt..

[60] Mar Blanca C., Saloma C. (1998). Monte Carlo analysis of two-photon fluorescence imaging through a scattering medium. Appl. Opt..

[61] Fales A.M., Vogt W.C., Wear K.A., Ilev I.K., Pfefer T.J. (2019). Pulsed laser damage of gold nanorods in turbid media and its impact on multi-spectral photoacoustic imaging. Biomed. Opt. Express.

[62] Okawa S., Hirasawa T., Sato R., Kushibiki T., Ishihara M., Teranishi T. (2018). Numerical and experimental investigations of dependence of photoacoustic signals from gold nanoparticles on the optical properties. Opt. Rev..

[63] Neuwelt A., Sidhu N., Hu C.A., Mlady G., Eberhardt S.C., Sillerud L.O. (2015). Iron-based superparamagnetic nanoparticle contrast agents for MRI of infection and inflammation. Am. J. Roentgenol..

[64] Albayedh F., Chow J.C. (2018). Monte Carlo simulation on the imaging contrast enhancement in nanoparticle-enhanced radiotherapy. J. Med. Phys..

[65] Ross G.M. (1999). Induction of cell death by radiotherapy. Endocr. Relat. Cancer.

[66] Xing J.L., Stea B. (2024). Molecular mechanisms of sensitivity and resistance to radiotherapy. Clin. Exp. Metastasis.

[67] Chen Y., Yang J., Fu S., Wu J. (2020). Gold nanoparticles as radiosensitizers in cancer radiotherapy. Int. J. Nanomed..

[68] Incerti S., Suerfu B., Xu J., Ivantchenko V., Mantero A., Brown J.M., Bernal M.A., Francis Z., Karamitros M., Tran H.N. (2016). Simulation of Auger electron emission from nanometer-size gold targets using the Geant4 Monte Carlo simulation toolkit. Nucl. Instrum. Methods Phys. Res. Sect. B Beam Interact. Mater. At..

[69] Rosero W.A., Barbezan A.B., de Souza C.D., Rostelato M.E. (2024). Review of advances in coating and functionalization of gold nanoparticles: From theory to biomedical application. Pharmaceutics.

[70] Cho S.H. (2005). Estimation of tumour dose enhancement due to gold nanoparticles during typical radiation treatments: A preliminary Monte Carlo study. Phys. Med. Biol..

[71] Dos Santos M., Delorme R., Salmon R., Prezado Y. (2020). Minibeam radiation therapy: A micro- and nano-dosimetry Monte Carlo study. Med. Phys..

[72] Sheeraz Z., Chow J.C.L. (2021). Evaluation of dose enhancement with gold nanoparticles in kilovoltage radiotherapy using the new EGS geometry library in Monte Carlo simulation. AIMS Biophys..

[73] Martinov M.P., Thomson R.M. (2017). Heterogeneous multiscale Monte Carlo simulations for gold nanoparticle radiosensitization. Med. Phys..

[74] Zheng X.J., Chow J.C. (2017). Radiation dose enhancement in skin therapy with nanoparticle addition: A Monte Carlo study on kilovoltage photon and megavoltage electron beams. World J. Radiol..

[75] Harriss-Phillips W.M., Bezak E., Yeoh E.K. (2011). Monte Carlo radiotherapy simulations of accelerated repopulation and reoxygenation for hypoxic head and neck cancer. Br. J. Radiol..

[76] Zerakni F., Dib A.S., Attili A. (2024). Enhancing tumor’s skin photothermal therapy using Gold nanoparticles: A Monte Carlo simulation. Lasers Med. Sci..

[77] Ma S., Jiang L., Yang W., Liu F., Wang D., Wang F., Huang J. (2024). Advances of Nanomaterials in Cancer Photocatalysis Therapy. Mater. Today Sustain..

[78] Maurente A., de Sousa A.N. (2024). Anisotropic scattering in photothermal therapy and beyond: A detailed evaluation of transport approximation for gold nanoshells and nanospheres. J. Braz. Soc. Mech. Sci. Eng..

[79] Periyasamy V., Pramanik M. (2017). Advances in Monte Carlo simulation for light propagation in tissue. IEEE Rev. Biomed. Eng..

[80] Jin W., Shi X., Yin H., Zhang H., Wang Z., Chen Q., Wu H., Han Y., Li Y. (2020). Comparison of actual and simulated tumoricidal effects induced by photodynamic therapy. Photodiagnosis Photodyn. Ther..

[81] Kim D., Paik J., Kim H. (2023). Effect of gold nanoparticles distribution radius on photothermal therapy efficacy. Sci. Rep..

[82] Hosseini F.S., Naghavi N., Sazgarnia A. (2023). A physicochemical model of X-ray induced photodynamic therapy (X-PDT) with an emphasis on tissue oxygen concentration and oxygenation. Sci. Rep..

[83] Bucharskaya A.B., Khlebtsov N.G., Khlebtsov B.N., Maslyakova G.N., Navolokin N.A., Genin V.D., Genina E.A., Tuchin V.V. (2022). Photothermal and photodynamic therapy of tumors with plasmonic nanoparticles: Challenges and prospects. Materials.

[84] Siddique S., Chow J.C.L. (2022). Recent advances in functionalized nanoparticles in cancer theranostics. Nanomaterials.

[85] Montenegro M., Nahar S.N., Pradhan A.K., Huang K., Yu Y. (2009). Monte Carlo simulations and atomic calculations for Auger processes in biomedical nanotheranostics. J. Phys. Chem. A.

[86] Lindstaedt A., Doroszuk J., Machnikowska A., Dziadosz A., Barski P., Raffa V., Witt D. (2024). Effects Induced by the Temperature and Chemical Environment on the Fluorescence of Water-Soluble Gold Nanoparticles Functionalized with a Perylene-Derivative Dye. Materials.

[87] Tian Y., Carrillo-Malani N., Feng K., Miller J., Busch T.M., Sundaram K.M., Cheng Z., Amirshaghaghi A., Tsourkas A. (2024). Theranostic phthalocyanine and naphthalocyanine Nanoparticles for photoacoustic imaging and photothermal therapy of tumors. Nanotheranostics.

[88] Herzog J.M., Sick V. (2024). MCRAD: A Monte Carlo photon transport code for analysis of fluorescence and elastic scattering diagnostics. SoftwareX.

[89] Díaz-Galindo C.A., Garnica-Garza H.M. (2024). Radiation source personalization for nanoparticle-enhanced radiotherapy using dynamic contrast-enhanced MRI in the treatment planning process. Radiat. Phys. Chem..

[90] Chow J.C.L., Jubran S. (2023). Depth Dose Enhancement in Orthovoltage Nanoparticle-enhanced Radiotherapy: A Monte Carlo Phantom Study. Micromachines.

[91] Cogno N., Bauer R., Durante M. (2024). Mechanistic model of radiotherapy-induced lung fibrosis using coupled 3D agent-based and Monte Carlo simulations. Commun. Med..

[92] Taheri A., Khandaker M.U., Moradi F., Bradley D.A. (2024). A simulation study on the radiosensitization properties of gold nanorods. Phys. Med. Biol..

[93] Rojas-Domínguez A., Arroyo-Duarte R., Rincón-Vieyra F., Alvarado-Mentado M. (2022). Modeling cancer immunoediting in tumor microenvironment with system characterization through the ising-model Hamiltonian. BMC Bioinform..

[94] Metzcar J., Wang Y., Heiland R., Macklin P. (2019). A review of cell-based computational modeling in cancer biology. JCO Clin. Cancer Inf..

[95] Mountain R.D., Thirumalai D. (1994). Quantative measure of efficiency of Monte Carlo simulations. Phys. A Stat. Mech. Its Appl..

[96] Luo C., Keshtegar B., Zhu S.P., Taylan O., Niu X.P. (2022). Hybrid enhanced Monte Carlo simulation coupled with advanced machine learning approach for accurate and efficient structural reliability analysis. Comput. Methods Appl. Mech. Eng..

[97] Chow J.C.L., Ruda H.E. (2024). Impact of Scattering Foil Composition on Electron Energy Distribution in a Clinical Linear Accelerator Modified for FLASH Radiotherapy: A Monte Carlo Study. Materials.

[98] Lai Y., Jia X., Chi Y. (2021). Modeling the effect of oxygen on the chemical stage of water radiolysis using GPU-based microscopic Monte Carlo simulations, with an application in FLASH radiotherapy. Phys. Med. Biol..

[99] Mazzola G. (2024). Quantum computing for chemistry and physics applications from a Monte Carlo perspective. J. Chem. Phys..

[100] Chow J.C.L. (2024). Quantum Computing in Medicine. Med. Sci..

[101] Neph R., Lyu Q., Huang Y., Yang Y.M., Sheng K. (2021). DeepMC: A deep learning method for efficient Monte Carlo beamlet dose calculation by predictive denoising in magnetic resonance-guided radiotherapy. Phys. Med. Biol..

[102] Gong F.Q., Xiong K., Cheng J. (2024). Constructing machine learning potential for metal nanoparticles of varying sizes via basin-hoping Monte Carlo and active learning. Natl. Sci. Open.

[103] Holmes J., Feng H., Zhang L., Fix M.K., Jiang S.B., Liu W. (2024). Fast Monte Carlo dose calculation in proton therapy. Phys. Med. Biol..

[104] O’Quigley J., Chevret S. (1991). Methods for dose finding studies in cancer clinical trials: A review and results of a Monte Carlo study. Stat. Med..

[105] Goldenholz D.M., Tharayil J., Moss R., Myers E., Theodore W.H. (2017). Monte Carlo simulations of randomized clinical trials in epilepsy. Ann. Clin. Transl. Neurol..

